# The Duration of Preoperative Administration of Single *α*-Receptor Blocker Phenoxybenzamine before Adrenalectomy for Pheochromocytoma: 18 Years of Clinical Experience from Nationwide High-Volume Center

**DOI:** 10.1155/2019/2613137

**Published:** 2019-11-16

**Authors:** Jie Tian, Zhengqing Bao, Yiming Yuan, Dong Fang, Yonghao Zhan, Tianyu Wang, Zheng Zhang, Zhou Liqun

**Affiliations:** ^1^Department of Urology, Peking University First Hospital, Beijing 100034, China; ^2^Institute of Urology, Peking University, Beijing 100034, China; ^3^National Urological Cancer Center, 100034 Beijing, China; ^4^Andrology Center, Peking University First Hospital, Beijing 100034, China; ^5^Department of Urology, The First Affiliated Hospital of Zhengzhou University, Zhengzhou 450003, China

## Abstract

**Purpose:**

There is no consensus for the optimum duration of preoperative administration of phenoxybenzamine (PXB) before adrenalectomy for pheochromocytoma. The aim of this study is to investigate whether perioperative hemodynamics and postoperative outcomes are related to the duration of PXB administration.

**Methods:**

In total, 102 patients managed preoperatively with single *α*-receptor blocker phenoxybenzamine were enrolled from 469 consecutive patients diagnosed histologically with pheochromocytoma. The patients received surgical treatment in the Department of Urology, Peking University First Hospital, between January 2001 and July 2018. All patients were divided into three groups: Group A (<14 d), Group B (14–30 d), and Group C (>30 d). Patient and tumor characteristics, intraoperative hemodynamics, and postoperative outcomes were recorded and compared among the three groups.

**Results:**

These patients included 47 men and 55 women, with an average age of 43 years at the time of surgery. Clinical characteristics, except the status of preoperative biochemical tests (24 hr urine fractioned catecholamine or plasma-fractioned catecholamine) (*p*=0.020), preoperative hemodynamics, and medicine management and surgical approaches, in the three groups were comparable. Multivariate analyses demonstrated that the size of the tumor (*p*=0.034) was an independent risk factor for intraoperative hemodynamic instability. Among the three groups, we found no significant difference in intraoperative hemodynamics and postoperative outcomes.

**Conclusion:**

The data from the current study indicated that the preoperative management of pheochromocytoma with single *α*-receptor blocker PXB for more than 2 weeks, after the final dose adjustment, could not further reduce the risk of intraoperative hemodynamic instability or postoperative complications. Thus, our study supports that 14 days would be enough for the duration of preoperative management of pheochromocytoma with single *α*-receptor blocker PXB in final dose.

## 1. Introduction

Pheochromocytoma, a rare catecholamine-producing tumor arises from chromaffin cells in the adrenal medulla and may cause a series of clinical manifestations, typically including hypertension, diaphoresis, and tachycardia [1, 2]. Currently, surgical resection is the only available curative treatment. Unfortunately, tumor manipulation during operation can trigger uncontrolled release of catecholamines that may lead to potentially lethal hypertensive crises and arrhythmias. In addition, this release of catecholamines can also be seen in patients with normotensive and asymptomatic tumors [3]. A group of researchers and authors found that antihypertensive drugs could lead to a reduction in perioperative complications from 69% to 3% based on their follow-up of 48 years [4]. Williams et al. [5] tested adequate preoperative pharmacological control of hypertension for 47 patients with pheochromocytoma. No one died after surgery, and only 8 patients had postoperative complications. Cure of hypertension was achieved in 80% of patients. The recommendation suggested that all patients with pheochromocytoma should receive appropriate preoperative medical management to block the effects of circulating catecholamines. Most commonly, phenoxybenzamine (PXB), a nonselective *α*-receptor blocker, is used for operative preparation [6]. At the First International Symposium on Pheochromocytoma (ISP2005), held in Bethesda in October 2005, a panel of experts were in general agreement that preoperative phenoxybenzamine management should last at least 1 week, doses should be patient-titrated, and treatment efficacy should be assessed mainly from the patient's blood pressure profile [7]. However, there is no consensus for the optimum duration of preoperative use of PXB. Prospective studies that analyze the impact of the duration of preoperative preparation with PXB on perioperative hemodynamic alterations and postoperative outcomes are lacking. Therefore, we conducted the present study to investigate whether perioperative hemodynamics and postoperative outcomes are related to the duration of single *α*-receptor blocker phenoxybenzamine.

## 2. Patients and Methods

### 2.1. Patients Enrollment and Evaluation

Between January 2001 and July 2018, 469 consecutive patients diagnosed histologically with pheochromocytoma received surgical treatment in the Department of Urology, Peking University First Hospital. In total, 367 patients were excluded from this study, including 297 patients who received others or more *α*-receptor blockers before surgery, 23 patients with bilateral or multiple pheochromocytomas, 44 patients with incomplete data, and 3 patients with extra-adrenal tumors. Finally, 102 patients were enrolled.

All patients experienced at least one catecholamine test before surgery, such as adrenaline, norepinephrine, dopamine for plasma or urinary, and vanillylmandelic acid. Since 2016, we have performed metanephrines testing for patients with suspected pheochromocytoma. Therefore, of the 102 patients in this group, only 12 patients were performed metanephrines testing. All patients' biochemical test values are considered to be positive beyond the upper limit of the normal range.

The initial dose of PXB was 10 mg orally twice daily, which would be increased daily until the blood pressure (BP) was controlled to no more than 140/80 mmHg and the peripheral circulation changes, the limbs touch the warmth. Patients can be slightly tolerant of side effects of phenoxybenzamine, such as orthostatic hypotension and nasal stiffness [1]. The duration of preoperative management, called duration of final dose (DFD), ranged from the time of final dose adjustment to surgery. Other hypertensive drugs such as angiotensin-converting enzyme inhibitors (ACEIs), angiotensin receptor blockers (ARBs), or calcium channel blockers (CCBs) were also considered when required. *β*-Blockers were administered to control tachycardia and supraventricular arrhythmia. All patients were prepared for surgery with intravenous saline infusion 1-2 L in the evening before surgery.

After preoperative management with single *α*-receptor blocker phenoxybenzamine, all patients underwent adrenalectomy under general anesthesia by experienced surgeons. All patients received an arterial line for blood pressure monitoring. Blood pressure measurements were automatically recorded every 10 seconds by computer. Intraoperative hemodynamic instability was defined as follows: (1) systolic blood pressure (SBP) > 200 mmHg; (2) SBP > 130% of basic SBP; (3) SBP < 80 mmHg; (4) SBP < 70% of basic SBP; (4) heart rate (HR) > 120 bpm. Basic SBP was defined as the SBP before preoperative management with PXB. In addition, the degree of instability was indicated by the total area of under the SBP-time curve (AUC) outside of the preset SBP range (80–200 mmHg and 70% of basic SBP–130% of basic SBP), standardized with per minute anesthesia time in mmHg × s·min^−1^.

According to DFD, 102 patients were divided into three groups: Group A (<14 d), Group B (14–30 d), and Group C (>30 d). Patient and tumor characteristics, intraoperative hemodynamics, and postoperative outcomes were recorded and compared among the three groups.

The primary outcome was intraoperative hemodynamic instability, and the secondary end point was postoperative complications. Complications directly related to CV hemodynamic stability included new arrhythmia and postoperative hypotension and use of vasopressor, and complications not directly related to CV hemodynamic stability included pulmonary complications, postoperative stroke, wound infection, intestinal obstruction, urinary tract infection, acute liver injury after operation, postoperative acute kidney injury, venous thrombosis, and hypoglycemia. The criteria of the Japan Clinical Oncology Group were used to evaluate the severity of the complications [8].

## 3. Statistical Analysis

All statistical tests were performed with SPSS 20.0 (IBM Corp, Armonk, NY, USA), and statistical significance was set at *p* < 0.05. Pearson's test and the chi-square test were used to test the distribution of categorical variables, and the *F*-test was used for continuous variables. The Kruskal–Wallis test was used for nonnormal distribution variables. Multivariate logistic regression was used to adjust the effect of other variables on intraoperative hemodynamics and postoperative outcomes.

## 4. Result

### 4.1. Patient and Tumor Characteristics

These patients included 47 men and 55 women, with average age of 43 years at the time of surgery. All patients were divided into three groups: 37 cases in Group A, 26 cases in Group B, and 39 cases in Group C. Clinical characteristics, except the status of preoperative biochemical tests (24 hr urine-fractioned catecholamine or plasma-fractioned catecholamine) (*p*=0.020), and surgical approaches in the three groups were comparable, as shown in Table 1. We also found no significant difference in preoperative hemodynamics and medicine management.

### 4.2. Intraoperative Hemodynamics and Postoperative Outcomes

The detailed data of Intraoperative hemodynamics are shown in Table 2. Among the three groups, we found no significant difference in intraoperative minimum SBP (*p*=0.688), intraoperative maximum SBP (*p*=0.400), or intraoperative maximum HR (*p*=0.321). Also no significant difference was noticed regarding the incidence of intraoperative minimum SBP < 80 mmHg, intraoperative maximum SBP > 200 mmHg, or intraoperative maximum HR > 120 bpm as well (*p*=0.606, 0.331 and 0.205, respectively). The AUCs outside of 70–130% basic SBP were 59.8 (19.1–149.5), 41.4 (18.1–123.6), and 78.8 (19.2–185.9) mmHg × s·min^−1^, respectively (*p*=0.445).

### 4.3. Complications

Finally, we found 38 people with 53 complications in our research. These complications are summarized in Table 3.

As shown in Table 4, the requirement of Intensive Care Unit (ICU), ventilator support, postoperative hypotension, complications, and postoperative hospitalization exhibited no significant difference (*p*=0.276, 0.118, 0.791, 0.140, and 0.773, respectively). Multivariate analyses demonstrated that the size of the tumor (*p*=0.034) was an independent risk factor for intraoperative hemodynamic instability (Table 5).

## 5. Discussion

Intraoperative hemodynamic instability significantly increases the risk of major morbidity for patients with pheochromocytomas [9]. Preoperative management with an adequate *α*-receptor blocker has been proven to decrease the risk of perioperative complications, including intraoperative hemodynamic crisis [2, 10]. Phenoxybenzamine, a nonselective *α*-antagonist, has been widely adopted to help minimize intraoperative hemodynamic instability and help control catecholamine fluctuations [6, 11].

However, the optimal duration of this therapy remains unclear [12]. Currently, the duration of preoperative management with PXB is based on previous experience and varies from institution to institution. The median duration of preoperative management with PXB in our center is 27.4 days. However, researchers have reported median duration of PXB for 16 days, 35 days, or even 14 weeks [3, 13, 14]. Prospective studies that analyze the impact of the duration of preoperative preparation with PXB on perioperative hemodynamic alterations and postoperative outcomes are lacking. Furthermore, even the definition of the duration of therapy remains unclear. Previous researchers defined the duration of preoperative management as the time ranged from the time of beginning to surgery. However, our study defined the duration as the time ranged from the time of final dose adjustment to surgery.

As for intraoperative hemodynamic instability, previous studies only focused on the change of intraoperative BP at one point, using the cutoff value of intraoperative SBP > 200 mmHg, SBP >160 mmHg, or SBP < 80 mmHg [13, 15, 16], and blood pressure was recorded over a long time interval. Therefore, the patient's blood pressure fluctuations cannot be fully evaluated, and the data for abnormal BP might be lost. In our study, BPs were automatically recorded every 10 seconds by computer, and we took the AUC as the evaluation indicator, which made our results more persuasive.

We found no significant difference in intraoperative hemodynamics and postoperative complications between the study groups, which is consistent with some previous studies [17, 18]. Our result indicated that preoperative management of pheochromocytoma with single *α*-receptor blocker PXB more than 2 weeks, after the final dose adjustment, could not reduce the risk of intraoperative hemodynamic instability or postoperative complications. Interestingly, there are studies showing that long-term preoperative medication seems unnecessary. We did not test the circulating catecholamine after the BP had been controlled, which would be extremely useful to guide the proper duration of preoperative *α*-blocker administration. The current study indicated that possibly the circulating catecholamine has been normalized or at least nonlethal. Boutros suggested that patients with pheochromocytoma can undergo successful surgery without preoperative management with *α*-receptor blockers [19]. However, these conclusions should be interpreted with caution, as relatively small groups of patients were enrolled. Recent guidelines recommended all patients, even normotensive, should receive a blockade preoperative treatment to prevent unpredictable increases in blood pressure during surgery.

Previous studies revealed that both the size of the tumor and degree of catecholamine production strongly correlated with intraoperative hemodynamic instability [20, 21]. Kiernan demonstrated that after open surgery, the risk of increased number of episodes of systolic blood pressure > 200 mmHg was 27 times higher compared to laparoscopy [14]. In our study, clinical characteristics, except the status of preoperative biochemical tests (*p*=0.020), and surgical approaches in the three groups were comparable, and we found no significant difference in preoperative hemodynamics and medicine management as well. All of these observations make our conclusion more reliable. In our study, only tumor size is correlated with intraoperative hemodynamic instability, which reminds us the necessity to pay special attention to those with large-sized tumors and special measures should be taken to avoid complications.

There are certainly limitations of the study. First, this study represents a retrospective review of data at a single center, which might be related to selective and recall bias, and further external validation (especially from non-Chinese cohorts) would be important. Second, our study did not take perioperative fluid usage into consideration. Last, since 2016, we have performed metanephrines testing for patients with suspected pheochromocytoma. Therefore, of the 102 patients in this group, only for 12 patients metanephrines testing was performed. Although all patients were positive for metanephrine test, this index could not be included in the risk factor analysis. Finally, there was no uniform protocol followed by the anesthetists to control intraoperative hemodynamic instability, and the conclusion needs a large sample of prospective randomized controlled studies for further verification.

## 6. Conclusion

The data from the current study indicated that preoperative management of pheochromocytoma with single *α*-receptor blocker PXB more than 2 weeks, after the final dose adjustment, could not further reduce the risk of intraoperative hemodynamic instability or postoperative complications. Thus, our study supports that 14 days would be enough for the duration of preoperative management of pheochromocytoma with single *α*-receptor blocker PXB in final dose.

## Figures and Tables

**Table 1:** Patient and tumor characteristics.

	Group A	Group B	Group C	Overall	*p*
No. of cases, *n*	37	26	39	102	
Age	41.54 ± 13.61	42.58 ± 15.15	44.87 ± 13.62	43.08 ± 13.96	0.574
Gender					0.171
Female	21 (56.8%)	10 (38.5%)	24 (61.5%)	55 (53.9%)	
Male	16 (43.2%)	16 (61.5%)	15 (38.5%)	47 (46.1%)	
BMI	23.32 ± 3.1	23.55 ± 3.89	23.83 ± 3.7	23.58 ± 3.52	0.824
Preoperative BP					
SBP	126.76 ± 13.73	121.35 ± 14.93	127.31 ± 15.31	125.59 ± 14.73	0.234
DBP	79 (69.5–86)	74 (66.75–82.75)	75 (70–83)	77 (68.75–85)	0.383
Preoperative HR	77 (71.5–80)	73 (70–78)	75 (70–79)	75.5 (70–80)	0.252
Preoperative BP_max_					
SBP_max_	190 (160–220)	180 (157.5–202.5)	190 (170–220)	190 (160–220)	0.321
DBP_max_	114.54 ± 20.67	102.77 ± 16.1	110.26 ± 26.25	109.9 ± 22.29	0.118
Surgical approach					0.093
Open	9 (24.3%)	12 (46.2%)	18 (46.2%)	39 (38.2%)	
Endoscopic	28 (75.7%)	14 (53.8%)	21 (53.8%)	63 (61.8%)	
Size of tumor, cm	4.5 (4.25–6.5)	5 (4.08–7.5)	6 (4.5–9)	5 (4.18–7.5)	0.253
Location of tumor					0.723
Left	18 (48.6%)	10 (38.5%)	17 (43.6%)	45 (44.1%)	
Right	19 (51.4%)	16 (61.5%)	22 (56.4%)	57 (55.9%)	
Biochemical positive	22 (59.5%)	20 (76.9%)	34 (87.2%)	76 (74.5%)	0.020^*∗*^
Average dose of PXB	29.17 (20.38–42.31)	26.74 (15–32.25)	30 (20–40)	29.4 (20–40)	0.415
Cases of *β*-blockers	11 (29.7%)	7 (26.9%)	13 (33.3%)	31 (30.4%)	0.854
Cases of ACEIs	3 (8.1%)	2 (7.7%)	2 (5.1%)	7 (6.9%)	0.893
Cases of ARBs	2 (5.4%)	0 (0.0%)	3 (7.7%)	5 (4.9%)	0.437
Cases of CCBs	11 (29.7%)	6 (23.1%)	11 (28.2%)	28 (27.5%)	0.836

BMI, body mass index; BP, blood pressure; SBP, systolic blood pressure; DBP, diastolic blood pressure; HR, heart rate; PXB, phenoxybenzamine (mg/d); ACEIs, angiotensin-converting enzyme inhibitors; ARBs, angiotensin receptor blockers; CCBs, calcium channel blockers. ^*∗*^Statistically significant.

**Table 2:** Intraoperative hemodynamics of the three groups.

	Group A	Group B	Group C	Overall	*p*
Intraoperative SBPmin	81.57 ± 11.29	78.42 ± 16.72	78.97 ± 18.98	79.77 ± 15.88	0.688
<80 mmHg, *n*	15 (40.5%)	13 (50.0%)	20 (51.3%)	48 (47.1%)	0.606
AUC^#^, mmHg × s·min^−1^	0 (0–1.4)	0.1 (0–5.5)	0.2 (0–4.5)	0 (0–4.03)	0.551
<70% of basic BP	24 (64.9%)	15 (57.7%)	24 (61.5%)	63 (61.8%)	0.846
AUC, mmHg × s·min^−1^	4.7 (0–19.6)	1.8 (0–14)	3.4 (0–83.1)	3.8 (0–27.9)	0.457
Intraoperative SBPmax	198 (183.5–216.0)	189.5 (174.25–203.5)	193 (172–212)	194 (175.7–211.2)	0.400
>200 mmHg, *n*	16 (43.2%)	7 (26.9%)	17 (43.6%)	40 (39.2%)	0.331
AUC, mmHg × s·min^−1^	0 (0–4.38)	0 (0–0.23)	0 (0–2.84)	0 (0–2.81)	0.423
>130% of basic BP	33 (89.2%)	23 (88.5%)	35 (89.7%)	91 (89.2%)	1.000
AUC, mmHg × s·min^−1^	33.4 (4.1–139.9)	25.3 (3.1–82.3)	10.2 (0.9–83.5)	19.0 (1.8–97.4)	0.529
Intraoperative HRmax	121.43 ± 16.09	115.81 ± 17.87	116.62 ± 16.2	118.16 ± 16.62	0.321
>120 bpm, *n*	21 (56.8%)	9 (34.6%)	17 (43.6%)	47 (46.1%)	0.205
AUC1	59.8 (19.1–149.5)	41.4 (18.1–123.6)	78.8 (19.2–185.9)	59.5 (19.1–156.9)	0.445
AUC2	2.0 (0–10.3)	0.4 (0–11.2)	2.8 (0–14.4)	1.6 (0–10.6)	0.700

^#^SBPmin, minimum systolic blood pressure; AUC, area under the SBP-time curve; SBPmax, maximum systolic blood pressure; HRmax, maximum heart rate; AUC1, AUC outside of 70–130% basic SBP; AUC2, AUC outside of 80–200 mmHg.

**Table 3:** Summary of postoperative complications based on the relationship with hemodynamics.

Variable	All complications *n* = 53
Directly related to CV hemodynamic stability	
New arrhythmia	3
Postoperative hypotension and use of vasopressor	30

Not directly related to CV hemodynamic stability	
Pulmonary complications	6
Postoperative stroke	2
Postoperative wound infection	2
Postoperative intestinal obstruction	2
Postoperative urinary tract infection	1
Acute liver injury after operation	2
Postoperative acute kidney injury	3
Venous thrombosis	1
Hypoglycemia	1

**Table 4:** Postoperative major outcome of the three groups.

	Group A	Group B	Group C	Overall	*p*
Cases of ICU needed, *n*	25 (67.6%)	21 (80.8%)	32 (82.1%)	78 (76.5%)	0.276
Ventilator support, *n*	14 (37.8%)	13 (50.0%)	24 (61.5%)	51 (50.0%)	0.118
Postoperative hypotension, *n*	10 (27.0%)	7 (26.9%)	13 (33.3%)	30 (29.4%)	0.791
Complications, *n*	10 (27%)	9 (34.6%)	19 (48.7%)	38 (37.3%)	0.140
Postoperative hospitalization, day	6 (4.5–7.5)	6 (3.75–9)	7 (4–9)	6 (4–8.25)	0.773

ICU, intensive care unit.

**Table 5:** Multivariate analyses of the correlations between DFD and intraoperative hemodynamics and postoperative outcome.

	Intraoperative hemodynamics		Postoperative complications	
	HR (95% CI)	*p*	HR (95% CI)	*p*
DFD<14 d	1.097 (0.365–3.298)	0.870	0.483 (0.169–1.379)	0.174
DFD ≥ 14 d	0.751 (0.236–2.392)	0.628	0.557 (0.192–1.617)	0.282
Age	1.019 (0.986–1.053)	0.252	0.990 (0.960–1.020)	0.512
Size of tumor	1.296 (1.020–1.674)	0.034^*∗*^	1.023 (0.872–1.201)	0.778
BMI	0.933 (0.821–1.059)	0.282	0.980 (0.870–1.105)	0.741
Surgical approach	1.872 (0.612–5.731)	0.272	1.454 (0.530–3.992)	0.467
Location of tumor	0.417 (0.165–1.053)	0.064	0.674 (0.268–1.560)	0.332
Biochemical positive	1.231 (0.412–3.674)	0.710	0.534 (0.179–1.598)	0.262

DFD, duration of final dose. ^*∗*^Statistically significant.

## Data Availability

The data sets of the current study are presented within additional supporting files. Some of them are available from the corresponding author on reasonable request.
